# Simultaneous degradation of two mycotoxins enabled by a fusion enzyme in food-grade recombinant *Kluyveromyces lactis*

**DOI:** 10.1186/s40643-021-00395-1

**Published:** 2021-07-15

**Authors:** Yu Xia, Zifeng Wu, Rui He, Yahui Gao, Yangyu Qiu, Qianqian Cheng, Xiaoyuan Ma, Zhouping Wang

**Affiliations:** 1 State Key Laboratory of Food Science and Technology, Jiangnan University, Wuxi, 214122 China; 2 School of Food Science and Technology, Jiangnan University, Wuxi, 214122 China; 3 Collaborative Innovation Center of Food Safety and Quality Control in Jiangsu Province, Jiangnan University, Wuxi, 214122 China

**Keywords:** Mycotoxins, Aflatoxin B_1_, Zearalenone, Fusion enzyme, Degradation, *Kluyveromyces lactis*, Food-grade

## Abstract

Aflatoxin B_1_ (AFB_1_) and zearalenone (ZEN) are two predominant mycotoxins ubiquitously found in corn, peanuts, and other grains, which pose a great threat to human health. Therefore, safe and effective methods for detoxification of these mycotoxins are urgently needed. To achieve simultaneous degradation of multiple mycotoxins, a fusion enzyme ZPF1 was constructed by linking zearalenone hydrolase and manganese peroxidase with a linker peptide GGGGS. This fusion enzyme was secretory expressed successfully in the newly constructed food-grade recombinant strain *Kluyveromyces lactis* GG799(pKLAC1-ZPF1), and was investigated with the mycotoxins degradation efficiency in two reaction systems. Results showed that both AFB_1_ and ZEN can be degraded by ZPF1 in reaction system 1 (70.0 mmol/L malonic buffer with 1.0 mmol/L MnSO_4_, 0.1 mmol/L H_2_O_2_, 5.0 µg/mL AFB_1_ and ZEN, respectively) with the ratios of 46.46% and 38.76%, respectively. In reaction system 2 (50.0 mmol/L Tris–HCl, with 5.0 µg/mL AFB_1_ and ZEN, respectively), AFB_1_ cannot be degraded while ZEN can be degraded with the ratio of 35.38%. To improve the degradation efficiency of these mycotoxins, optimization of the induction and degradation conditions were fulfilled subsequently. The degradation ratios of AFB_1_ and ZEN by ZPF1 in reaction system 1 reached 64.11% ± 2.93% and 46.21% ± 3.17%, respectively. While in reaction system 2, ZEN was degraded by ZPF1 at a ratio of 41.45% ± 3.34%. The increases of degradation ratios for AFB_1_ and ZEN in reaction system 1 were 17.65% and 7.45%, respectively, while that for ZEN in reaction system 2 was 6.07%, compared with the unoptimized results.

**Supplementary Information:**

The online version contains supplementary material available at 10.1186/s40643-021-00395-1.

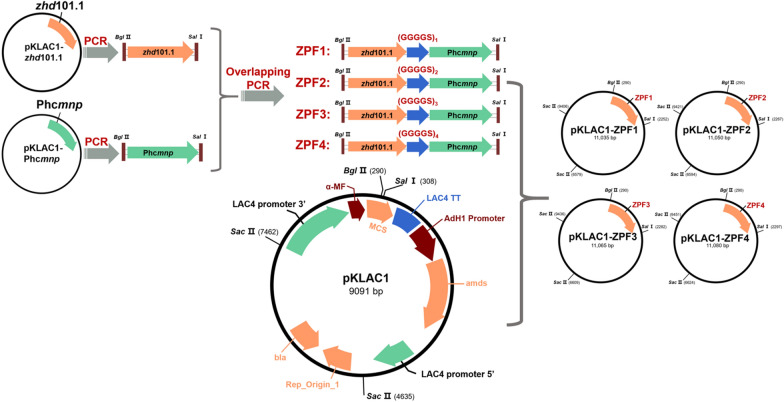

## Introduction

Among the mycotoxins that have been widely researched, aflatoxins (AFs) are the most toxic and pose the greatest threat to human health. Among all the AFs analogs, aflatoxin B_1_ (AFB_1_) possesses the strongest toxicity and usually causes severe harm to human beings and animals. For example, AFB_1_ is clearly associated to liver carcinogenesis, neurotoxicity, and growth retardation (Nazhand et al. 2020). Zearalenone (ZEN) is another kind of mycotoxins mainly caused by *Fusarium*, which is commonly found in corn and rice in high temperature or warm climate regions (Lee and Ryu 2017). ZEN contains estrogen-active isophthalic acid lactone, which can bind to and activate estrogen receptors, and disrupts the reproductive and endocrine systems. In general, most of the contaminated foods contains more than one kind of mycotoxins, and the interaction of multiple mycotoxins may cause more lethal damage (Lee and Ryu 2017; Alassane-Kpembi et al. 2017). With the presence of ZEN, AFB_1_ has a synergistic effect on cytotoxicity in a concentration-dependent manner. Low-level contaminated AFB_1_ can antagonize ZEN, but high-dose AFB_1_ and ZEN show a synergistic effect on oxidative damage (Lei et al. 2013).

Because of the harmful effects of mycotoxins to human health, efficient detoxification methods are urgently needed worldwide (Haque et al. 2020). For detoxification and degradation of mycotoxins, several kinds of strategies can be employed, such as physical, chemical, and biological methods (Nazhand et al. 2020; Liu et al. 2020; Wan et al. 2020; Wu et al. 2020; Zhou et al. 2020). Among these methods, biological detoxification is considered as the safer, gentler, and more effective choice for degradation of mycotoxins into less toxic intermediates or non-toxic products (Adebo et al. 2015; Wang et al. 2019a). It has been reported that a wide range of microorganisms including bacteria and fungi can decrease the toxicity of mycotoxins by cell wall adsorptions or intracellular metabolic processes (Armando et al. 2012; Brana et al. 2017; Gonzalez Pereyra et al. 2020; Zhang et al. 2021). Recently, the works of enrichment, purification, genetic engineering, and heterologous expression of many specific toxin-degrading enzymes are increasingly playing important roles in mycotoxins degradations (Loi et al. 2020; Wang et al. 2019b; Zhang et al. 2020). Considering the diversity of multiple mycotoxins contaminated in foods, a single specific mycotoxin-degrading enzyme can no longer meet the future needs. Although some probiotics, enzymes, and strains can be combined together to achieve simultaneous degradation of multiple mycotoxins, these methods are cumbersome, inefficient and not suitable for industrial applications (Afshar et al. 2020; Wang 2017; Yin 2018). Therefore, multifunctional enzymes that can simultaneously detoxify a wide range of mycotoxins are urgently needed.

Fusion protein technology is now widely used in field of protein engineering. Yet fusion enzymes for simultaneous degradation of multiple mycotoxins were seldom reported (Azam et al. 2019). Construction of fusion proteins involved in the linking of different proteins or domains by peptide linkers (Arai et al. 2001; Chen et al. 2013; Crasto and Feng 2000). Recently, the hydrolase ZHD101 and carboxypeptidase had been fused together with a connecting peptide containing five amino acids, and the fusion enzyme was successfully expressed in *Escherichia coli* with dual functions of degrading zearalenone (ZEN) and ochratoxin A (OTA) (Azam et al. 2019), which might be the first report of fusion protein used for mycotoxins degradation. However, *E. coli* is a type of opportunistic pathogen and is sometimes unsuitable for expression of fungal-derived enzymes, especially those enzymes used in food industry. In contrast, the food-grade yeast expression host *Kluyveromyces lactis* is the preferred one for expression of varieties of enzymes which involved in food processing (Lachance 2007; Pacheco et al. 2012; Wang et al. 2016).

In this work, a fusion enzyme of zearalenone hydrolase and manganese peroxidase for the simultaneous degradation of AFB_1_ and ZEN was designed, constructed and expressed in a food-grade manner. The zearalenone hydrolase ZHD101.1 (ZHD101 mutant V153H) from *Clonostachys rosea* (Xu et al. 2016; Xu 2019; Zhou 2017) and manganese peroxidase PhcMnp from *Phanerochaete chrysosporium* (Sun 2020; Jarvinen et al. 2012; Stewart et al. 1996; Coconi-Linares et al. 2014) were selected for construction of fusion enzymes, with (GGGGS)_n_ (*n* = 1 ~ 4) as the linker peptides. These two individual enzymes had ever been, respectively, expressed in *E. coli* (Hui et al. 2017; Whitwam et al. 1995) and yeasts (Gu et al. 2003; Xu 2019; Wang et al. 2020;). This fusion enzyme was successfully secretory expressed in the food-grade host *K. lactis* GG799. The degradation efficiency for AFB_1_ and ZEN by the fusion enzyme were evaluated, and the induction conditions, as well as the degradation conditions, were also optimized in this work.

## Materials and methods

### Chemicals and reaction systems

AFB_1_ and ZEN were purchased from Pribolab (Shandong, China). In this research work, these standards were made into a 1.0 mg/mL stock solution with methanol or acetonitrile solvent, and were preserved with refrigeration in darkness at − 20 ℃. Restriction enzymes, T4 DNA ligase, and plasmid extraction kits were purchased from Thermo Fisher Scientific (Shanghai, China). Hemin and sorbitol were purchased from Aladdin (Shanghai, China). Protein markers, protein loading buffers, SDS gel stain washing buffers, fungus genomic DNA extraction kits were purchased from Sangon Biotech (Shanghai, China). Methanol, formic acid, and acetonitrile were purchased from Tedia (OH, USA). All other reagents and chemicals were of analytical reagent grade.

Two reaction systems were used for mycotoxins degradation in this work. The reaction system 1 contained 70.0 mmol/L malonic acid buffer with 1.0 mmol/L MnSO_4_, 0.1 mmol/L H_2_O_2_, 5.0 µg/mL AFB_1_ and 5.0 µg/mL ZEN (Wang et al. 2019b). The reaction system 2 contained 50.0 mmol/L Tris–HCl (pH 7.5) with 5.0 µg/mL AFB_1_ and 5.0 µg/mL ZEN (Xiang et al. 2016).

### Plasmids, strains, cultural conditions and DNA manipulations

The coding genes for the enzymes ZHD101.1 and PhcMnp were previously synthesized and cloned into DH5α(pKLAC1-*zhd*101.1) and DH5α(pKLAC1-Phc*mnp*) by GenScript (Nanjing, China) and preserved in this laboratory. The expression hosts *K. lactis* GG799, *E. coli* DH5α and the secretory expression vector pKLAC1 were preserved in this laboratory. The plasmids pKLAC1-ZPF1, pKLAC1-ZPF2, pKLAC1-ZPF3, pKLAC1-ZPF4, and recombinant strain *K. lactis* GG799(pKLAC1-ZPF1) were constructed in this work.

Growth media components were purchased from Oxoid (Thermo Fisher Scientific, Shanghai). For cultivation of *K. lactis* GG799, the strain was grown in YEPD medium (yeast extract 1%, peptone 2%, glucose 2%, pH 6.3) at 30 ℃, 200 rpm for 18 to 22 h. For induction expression of ZPF1, YEPG medium (1.0% yeast extract, 2.0% peptone, 2.0% galactose, 0.5 mmol/L MnSO_4_, 0.5 mmol/L hemin, pH 5.9) was used and the strain was cultivated at 30 ℃, 200 rpm for 96 h. The genome DNA of *K. lactis* recombinants were extracted and purified by fungus genomic DNA extraction kit. All the DNA sequencing works were done in Sangon Biotech (Shanghai, China).

### Construction of fusion genes and recombinant plasmids

**Fig. 1:**
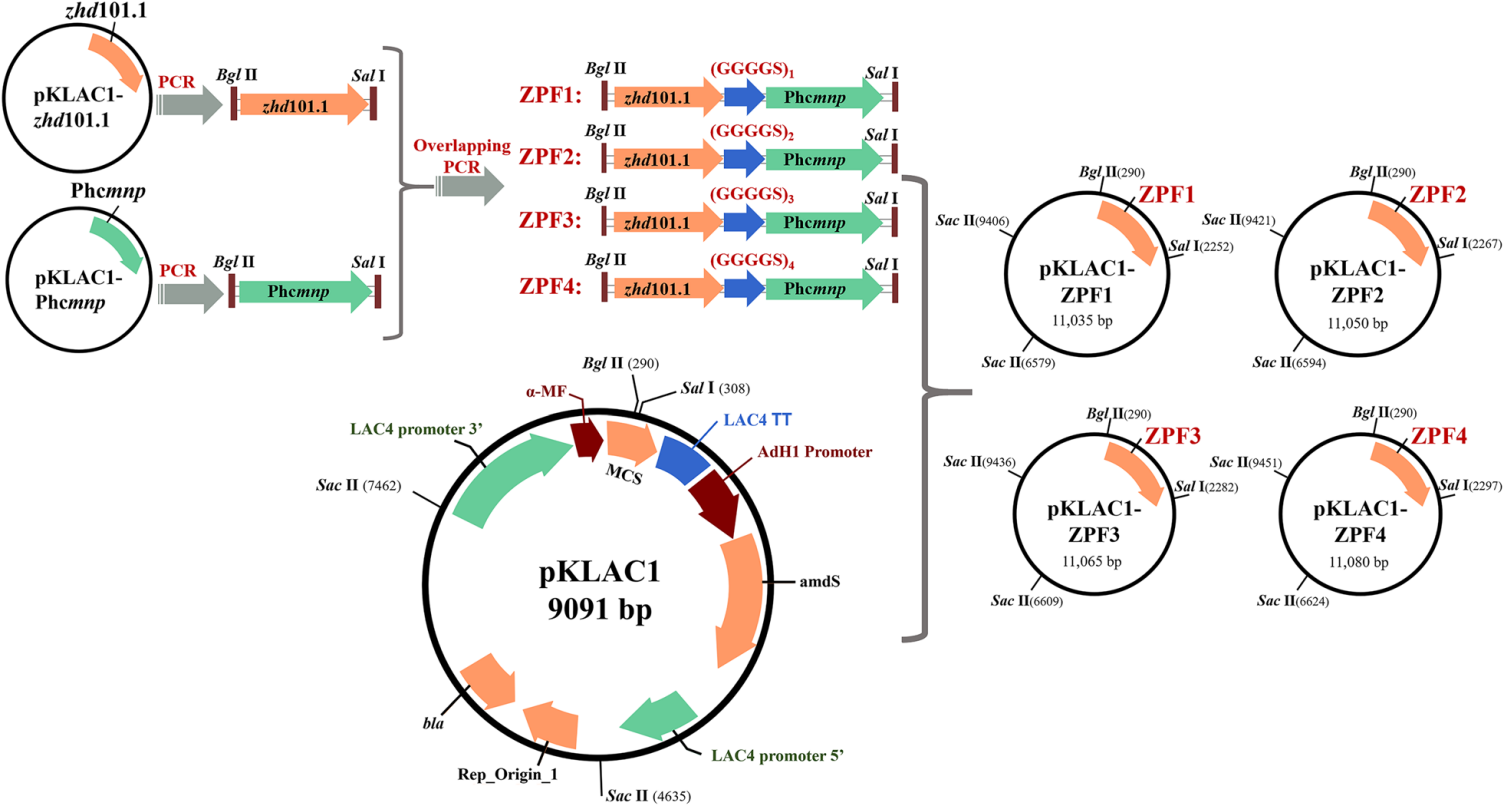
Flowchart for construction of fusion genes and recombinant plasmids

Plasmids in two strains DH5α(pKLAC1-*zhd*101.1) and DH5α(pKLAC1-Phc*mnp*) were extracted as templates. The primers (*zhd*101.1-F, *zhd*101.1-R) and (Phc*mnp*-F, Phc*mnp*-R) were used for PCR amplification of the two genes. The two PCR results were purified, respectively. These PCR results were mixed as templates, and the primers *zhd*101.1-Fn and Phc*mnp*-Rn (*n* = 1 ~ 4) were used for the overlapping PCR to obtain the fusion genes. The fusion genes obtained were then cloned in the expression vector pKLAC1 to generate recombinant plasmids pKLAC1-ZPFn. The recombinant plasmids from the positive clones were purified, verified by DNA sequencing and then transformed *K. lactis* GG799. The GG799 transformants were verified by PCR using the primers pKLAC1-seq-F and pKLAC1-seq-R. All the primers used for plasmids construction and verification are listed in Additional file 1: Table S1. Detailed procedures for construction of the plasmids and the recombinant strains are shown in Fig. 1.

### Expression of fusion enzyme ZPF1 in recombinant *K. lactis* GG799(pKLAC1-ZPF1)

The recombinants of *K. lactis* GG799(pKLAC1-ZPF1) were cultivated in the YEPD medium at 30 ℃ for about 18 to 22 h at a speed of 200 rpm. When the OD_600_ of the cultures reached 1.0, the cells were transferred into YEPG induction medium and cultivated at 30 ℃, 200 rpm for 96 h for expression of the fusion enzymes. The supernatants of these cultures were collected by centrifugation, and the protein samples were concentrated with 10.0-kDa ultrafiltration tubes. The concentrated solution samples containing the enzyme were used for subsequent biochemical analysis. Proteins were quantified with BCA protein quantitative kit, and then analyzed and identified by SDS-PAGE (20.0 μg proteins per lane).

### Degradation of AFB_1_ and ZEN by ZPF1

Since the two domains (ZHD101.1 and PhcMnp) on the fusion enzyme ZPF1 had different reaction requirements for degradation of the two mycotoxins, two different reaction systems were required to verify the AFB_1_ and ZEN degradation efficiency by ZPF1. The reaction system 1 and 2 mentioned above were employed for this purpose. For degradation of the two mycotoxins in reaction system 1, the solution containing all the samples needed was incubated at 30 ℃ for 9 h, followed by adding 3.0 mL methanol for termination of the reaction (Wang et al. 2019b). For degradation of the two mycotoxins in reaction system 2, the solution containing all the samples needed was incubated at 37 ℃ for 30 min, followed by adding 1.0 mL methanol for termination of the reaction (Xiang et al. 2016).

In the figures, the degradation ratios of ZEN in the reaction systems 1 and 2 were denoted as ZEN-1 and ZEN-2, respectively. The AFB_1_ and ZEN standards were prepared with the concentration gradients (0.1 μg/mL, 0.2 μg/mL, 0.5 μg/mL, 1.0 μg/mL, 2.0 μg/mL, 5.0 μg/mL, in acetonitrile) for determination of the standard curves. As controls of degradation reactions, the AFB_1_ and ZEN were prepared in buffers at the final concentration of 5.0 µg/mL, respectively. All the experiments were performed in triplicate.

### Quantitative analysis of AFB_1_ and ZEN

The reaction mixture was filtered through a 0.22-µm filter and the remaining contents of AFB_1_ and ZEN were analyzed by UPLC–MS (Sun 2020). The UPLC–MS running parameters were as following: chromatographic was done using ACQUITY UPLC® BEH C18 (2.1 × 50 mm, 1.7 µm particle size); the mobile phase was acetonitrile/water/formic acid; flow rate was 0.3 mL/min; column temperature was 40 ℃. Mass spectrometry parameters were as following: electrospray ion source; multiple reaction monitoring modes (MRM); cone Voltage was 3.0 kV; heating gas temperature was 500 ℃; ion source temperature was 150 ℃; desolventizing gas rate was 800.0 L/h.

### Enzyme activity assay

The fusion enzyme ZPF1 contained two enzyme domains (ZHD101.1 and PhcMnp). The enzyme activity of PhcMnp was determined by the phenol red method (Wang et al. 2014) as following: 10.0 mmol/L MnSO_4_, 100.0 mmol/L sodium malonate buffer (pH 4.5), 0.25 mmol/L phenol red and 1.0 mL crude enzyme. The absorbance of the reaction mixture was measured at 624 nm. The reaction was initiated by adding 100.0 mmol/L H_2_O_2_ and reacted for 5 min at 30 ℃. Then the reaction was immediately terminated by adding 1% NaOH to the system. The changes of absorbance before and after the reaction were calculated for determination of enzyme activities. One unit of enzyme activity (U) was defined as following: The quantity of enzyme PhcMnp needed for oxidation of 1.0 mmol of the substrate in 1 min. The enzyme activity of zearalenone hydrolase was characterized by the degradation ratio of ZEN (Zhang et al. 2020).

### Optimization of ZPF1 induction conditions

According to the previous researches, the promoter LAC4 in plasmid pKLAC1 was induced by galactose and inhibited by glucose (Xu 2019). The recombinant strain *K. lactis* GG799(pKLAC1-ZPF1) was cultivated with different induction conditions (induction time: 24 to 144 h, induction temperature: 15 ℃ to 35 ℃, concentration of galactose: 5.0 to 80.0 g/L, concentration of MnSO_4_: 0.1 to 5.0 mmol/L, and concentration of hemin: 0.05 to 2.0 mmol/L), and the enzymes were secretory expressed into the supernatants (Gnanamani et al. 2006). The expression levels of ZPF1 were single-factor optimized and characterized by specific enzyme activities (AFB_1_) or degradation ratios (ZEN).

### Optimization of mycotoxins degradation conditions

The AFB_1_ and ZEN degradation efficiency were optimized, respectively by testing of the degradation ratios at different reaction conditions. For optimization of AFB_1_ and ZEN degradation in reaction system 1, the reaction time (0.5 to 10 h), temperature (20 ℃ to 70 ℃), protein concentration (0.1 to 5.0 mg/mL), pH (3.0 to 5.5), concentration of MnSO_4_ (0.1 to 5.0 mmol/L), concentration of H_2_O_2_ (0.1 to 5.0 mmol/L) were investigated separately. For optimization of ZEN degradation in reaction system 2, the reaction time (10 to 180 min), temperature (20 ℃ to 70 ℃), protein concentration (0.1 to 5.0 mg/mL), pH (6.0 to 8.5) were also investigated separately. The detailed reaction procedures were performed according to the methods described above.

## Results and discussion

### Construction, expression and degradation of AFB_1_ and ZEN by ZPF1

**Fig. 2:**
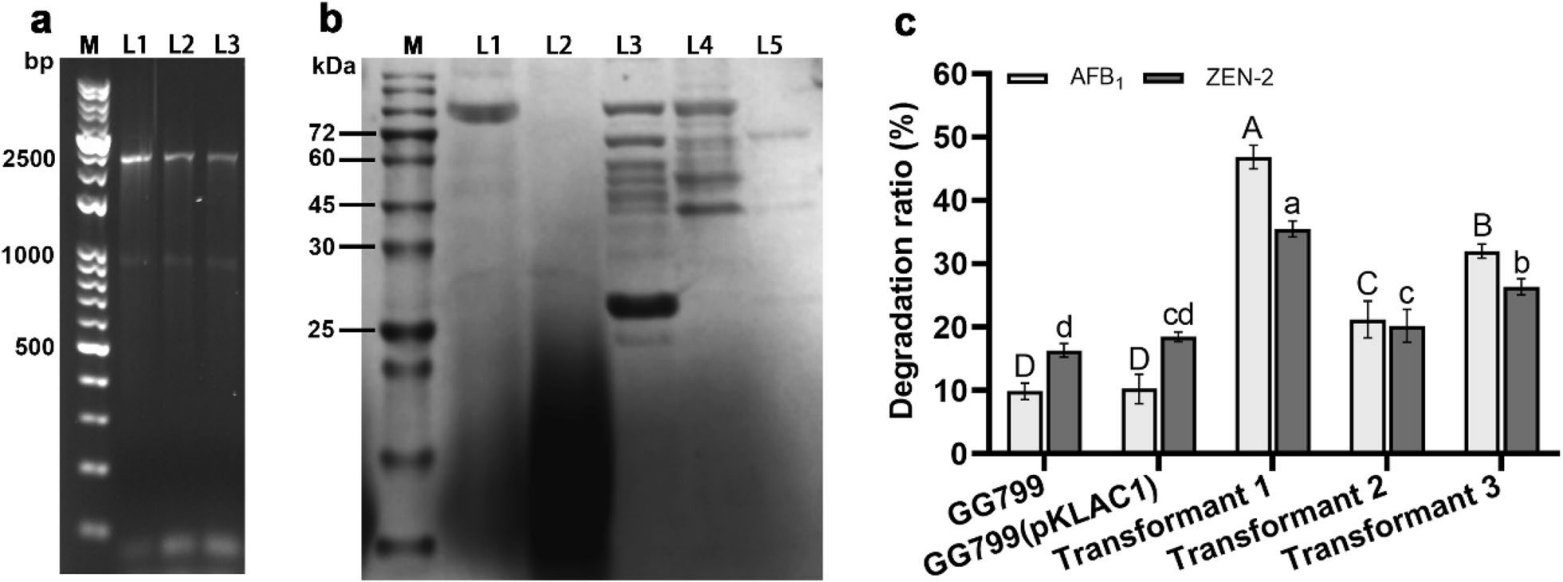
Construction and expression of fusion enzyme ZPF1, and the degradation efficiency of AFB_1_ and ZEN by ZFP1. **a** The electrophoresis of the PCR results of the 3 positive transformants: M: DNA marker; L1 to L3: PCR results of 3 positive GG799(pKLAC1-ZPF1) transformants. **b** SDS-PAGE results of culture supernatants of the 3 positive transformants: M: Protein marker; L1: *K. lactis* GG799 supernatant proteins; L2: *K. lactis* GG799(pKLAC1) supernatant proteins; L3 to L5: Three *K. lactis* GG799(pKLAC1-ZPF1) transformants’ supernatant protein. **c** Degradation results of AFB_1_ (reaction system 1) and ZEN (reaction system 2) by the 3 transformants. Different letters above the histogram are significant differences (*p* < 0.05)

To obtain recombinant fusion enzyme that can degrade AFB_1_ and ZEN simultaneously, four fusion plasmids were constructed in this work according to the methods described above. The flowchart for the construction is shown in Fig. 1. The recombinant plasmids were transferred *K. lactis* GG799 host, and the transformants were selected and verified by PCR amplifications. In these transformants, 3 positive transformants from the pKLAC1-ZPF1 transformation plates were successfully obtained. Other transformants from pKLAC1-ZPF2 to pKLAC1-ZPF4 transformation plates were all PCR negative. The PCR results of pKLAC1-ZPF1 transformants are shown in Fig. 2a. The lengths of the result bands were all around 2500 bp (Fig. 2a, Lane L1 to L3), which were near the theoretical size of the heterologous insertion. The PCR results were sequenced for determination of the successful constructions.

The 3 recombinant *K. lactis* GG799(pKLAC1-ZPF1) strains were induced and the enzymes were secretory expressed. As shown in the lane L3 in Fig. 2b, the expressed ZPF1 enzymes had the molecular weight of about 70.0 kDa on SDS-PAGE, which was close to its theoretical molecular weight of 69.23 kDa, while the controls (Fig. 2b, Lanes L1 and L2) showed no band around the target size. In comparison, the target protein in Lane L3 indicated the highest expression level among the 3 samples (Lanes L3 to L5). Besides, a protein band with the molecular weight above 25.0 kDa is found in Lane L3 in Fig. 2b. In consideration that the enzyme ZHD101.1 had the molecular weight of 28.8 KDa, this band might be the fractured domain of ZHD101.1 from the ZPF1.

The enzyme activities of PhcMnp and ZHD101.1 in the ZPF1 were determined according to the methods described above. The transformants 1, 2 and 3 showed the PhcMnp activities with the specific form of 3.18 U/mg, 0.94 U/mg, and 1.85 U/mg, respectively, while the enzyme activities of ZHD101.1 (characterized by ZEN degradation ratio) were 35.38%, 20.23% and 26.34%, respectively. As shown in Fig. 2c, the mycotoxins degradation efficiency using ZPF1 expressed by the 3 different transformants were investigated. Results showed that the enzyme from the transformant 1 had the best reaction activities to both AFB_1_ (in reaction system 1) and ZEN (in reaction system 2), with the degradation ratios of 46.46% and 35.38%, respectively (Fig. 2c). In contrast, the two negative controls, *K. lactis* GG799 and *K. lactis* GG799(pKLAC1) showed a small degradation efficiency of AFB_1_ and ZEN (about 10% and 17%, respectively). In conclusion, the transformant 1 showed the best performance in enzyme activity and degradation efficiency, and it was selected out for the further optimization.

### Optimization of induction expression conditions for ZPF1

**Fig. 3:**
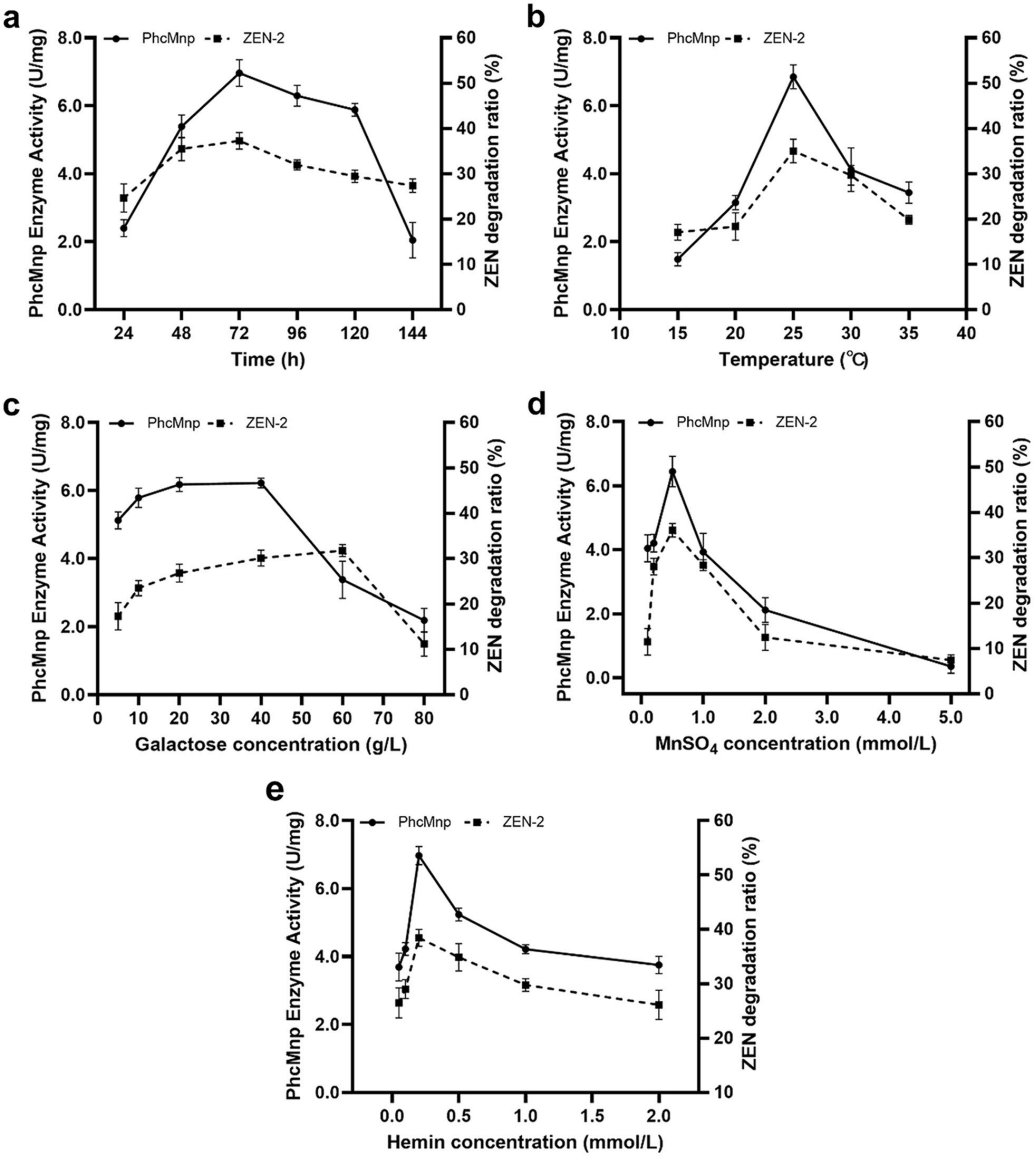
Enzyme activity of PhcMnp and ZHD101.1 expressed by ZPF1 under different induction conditions. The left axes were the PhcMnp enzyme activities, and the right axes were the degradation ratios of ZEN (by ZHD101.1). **a–e** were the effects of time, temperature, galactose concentrations, MnSO_4_ concentrations and hemin concentrations on the enzyme activities of the ZPF1

Enzyme expression, such as the expression of zearalenone hydrolase (Xu 2019), is usually influenced by several factors like incubation times, temperatures, and nitrogen source levels. In this system, the transcription and expression of the target enzyme were influenced by the concentration of galactose added. Besides, the PhcMnp activity of the ZPF1 was additional regulated by Mn^2+^ and hemin levels, during the induction expression procedure (Whitwam and Tien 1996; Gnanamani et al. 2006). Therefore, all these factors were investigated in this work, according to the methods described. As shown in Fig. 3, the trends of enzyme activities of PhcMnp and ZHD101.1 in ZPF1 under different induction conditions were approximately similar. With the increase of induction time (Fig. 3a), temperature (Fig. 3b), galactose (Fig. 3c), MnSO_4_ (Fig. 3d), and hemin (Fig. 3e) concentrations, the enzyme activities of both enzymes showed the trends of increasing and then decreasing.

It can be seen from Fig. 3c, a high concentration of galactose inhibited the fusion enzyme activity. This conclusion was similar to that from the previous research results. The galactose as an inducer added to the culture in low concentrations could not completely initiate heterologous protein expression, while high concentrations of it inhibited the protein expression, which might because of the carbon-to-nitrogen ratio (C/N) was not suitable any more (Xu 2019; Sun 2020).

As shown in Fig. 3d, the enzymatic activity gradually decreased as the Mn^2+^ concentration increased. When the concentration of Mn^2+^ was 0.5 mmol/L, the PhcMnp activity of the ZPF1 reached the highest value of 6.44 U/mg. With the increased concentration of Mn^2+^ added in the induction system, the PhcMnp activity decreased drastically. It was reported that enzyme PhcMnp was regulated by Mn^2+^ at the transcriptional level, and a certain amount of Mn^2+^ may promote the synthesis of PhcMnp (Wang 2014; Whitwam and Tien 1996). However, when the concentration of Mn^2+^ was too high, it inhibited the enzyme expression (Tang et al. 2012).

Furthermore, since PhcMnp is a glycosylated peroxidase containing heme iron (heme-Fe), which is essentially a highly helical pentameric protein containing Fe^3+^, additional appropriate amounts of hemin adding in the induction medium can provide Fe^3+^ for effectively initiation of enzyme synthesis (Jiang et al. 2008; Wang et al. 2016; Whitwam et al. 1995; Mayfield et al. 1994). However, similar to the influence of Mn^2+^ to the enzyme activity, high concentrations of hemin also inhibit PhcMnp enzyme activity (Fig. 3e), which is consistent with the findings of the previous reports (Jiang et al. 2008; Sun 2020).

Unlike the special requirements for expression of PhcMnp, the expression of ZHD101.1 was theoretically not affected by Mn^2+^ and hemin. However, high concentrations of Mn^2+^ also affects the enzyme expression by inhibiting the growth of yeast cells (Tang et al. 2012), so the enzyme activities of ZHD101.1 also showed decreasing trends with the increasing concentrations of Mn^2+^ added (Fig. 3d, e).

From the results shown above, it can be found that the optimized conditions for induction expression of ZPF1 were: inducing at 25 ℃ for 72 h, in YEPG medium containing 40.0 g/L galactose, 0.5 mmol/L MnSO_4_ and 0.2 mmol/L hemin.

### Optimization of degradation reaction conditions for AFB_1_ and ZEN

In this work, there were two systems for the degradation reaction of AFB_1_ and ZEN. The reaction system 1 contained MnSO_4_ and H_2_O_2_ and was used for degradation of AFB_1_ by PhcMnp, while the reaction system 2 was used for degradation of ZEN by ZHD101.1, as described in the methods above. Here the degradation conditions were optimized, respectively, in these two reaction systems.

**Fig. 4:**
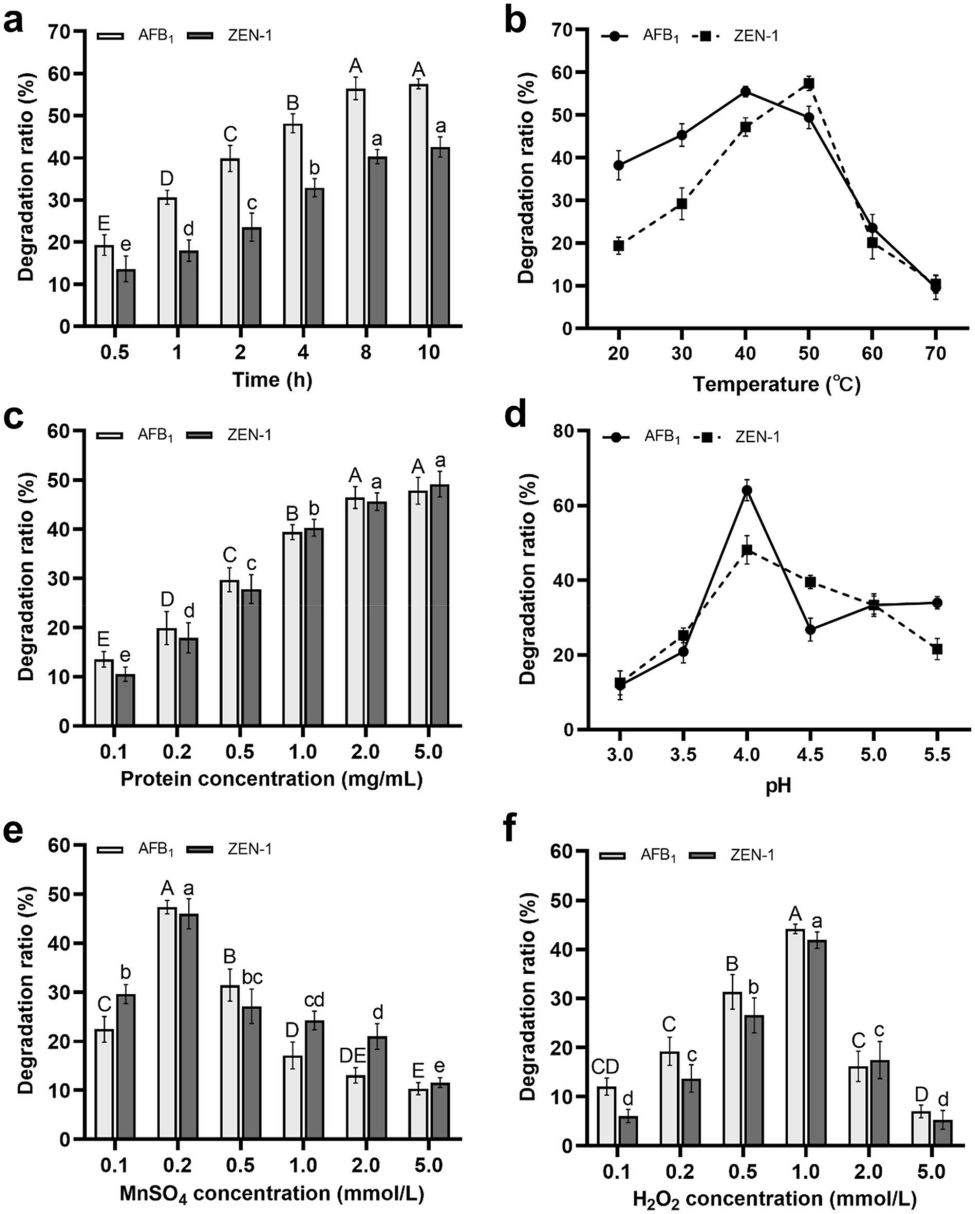
Degradation of AFB_1_ and ZEN by ZPF1 under different conditions in reaction system 1. **a–f** were the effects of time, temperature, protein concentrations, pH of buffer, concentrations of MnSO_4_ and concentrations of H_2_O_2_. Different letters above the histogram were significant differences (*p* < 0.05)

In reaction system 1, the conditions such as times, temperatures. protein concentrations and pH were investigated, respectively, and the results are shown in Fig. 4a–d. In this reaction system, the Mn^2+^ and H_2_O_2_ were critical elements for catalytic reactions. With the participation of a suitable concentration of H_2_O_2_, the Mn^2+^ was oxidized to Mn^3+^ by PhcMnp, and then the high redox potential of Mn^3+^ catalyzed and transferred the substrates to low toxic or non-toxic compounds (Whitwam and Tien 1996).

However, as shown in Fig. 4e, high concentrations of Mn^2+^ inhibited the degradation ratios of both AFB_1_ and ZEN. When the MnSO_4_ concentration was 0.2 mmol/L, the degradation ratios of AFB_1_ and ZEN were 47.36% and 46.01%, respectively. At the MnSO_4_ concentration of 5.0 mmol/L, the degradation ratios of AFB_1_ and ZEN were decreased to 10.35% and 11.6%. Furthermore, the degradation efficiency of mycotoxins was also affected by the concentration of H_2_O_2_ in reaction system 1. According to the previous reports, peroxidases could be inactivated through their own suicide inactivation mechanism with presence of high concentration of H_2_O_2_ (Valderrama et al. 2002; Zhang 2010). As shown in Fig. 4f, the degradation ratios of AFB_1_ and ZEN reached the maximum values (44.2% and 41.87%) at the H_2_O_2_ concentration of 1.0 mmol/L, while those at the H_2_O_2_ concentration of 5.0 mmol/L were 7.0% and 5.3%, respectively.

In conclusion, the optimal conditions for degradation of both mycotoxins by the ZPF1 in reaction system 1 were: malonic acid buffer (pH 4.0) containing 0.2 mmol/L MnSO_4_ and 1.0 mmol/L H_2_O_2_, added with 2.0 mg/mL expressed proteins, reacted at 40 ℃ for 8 h. Under optimized conditions, the degradation ratios of AFB_1_ and ZEN both reached the maximum data of 64.11% ± 2.93% and 46.21% ± 3.17%, respectively.

As for degradation of ZEN, most of the reported reaction systems for zearalenone hydrolases were Tris–HCl buffers (pH 7.5) (Xu 2019; Xiang et al. 2016), and this reaction system was used here as the reaction system 2 in this work. As shown by the results in Additional file 1: Figure S3, AFB_1_ cannot be degraded in this system, so here the optimal conditions were investigated for degradation of ZEN.

**Fig. 5:**
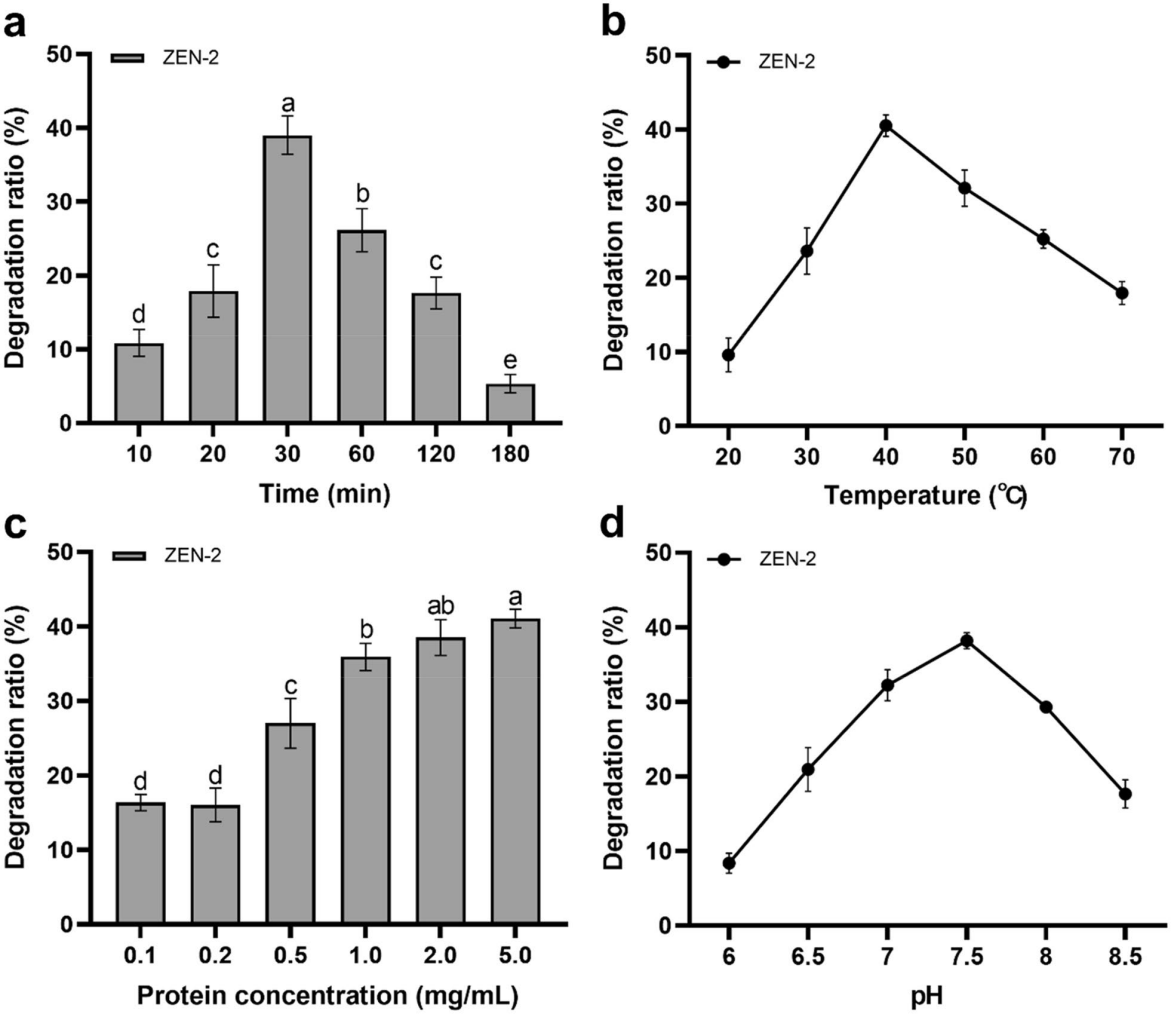
Degradation of ZEN by ZPF1 under different conditions in reaction system 2. **a–d** were the effects of time, temperature, protein concentrations and pH of buffer. Different letters above the histogram were significant differences (*p* < 0.05)

The degradation ratio for ZEN by ZPF1 reached 39.01% after 30-min reaction in reaction system 2 (Fig. 5a). In comparison, with the same reaction time (30 min), the degradation ratio of ZEN by ZPF1 in reaction system 1 was only 13.63% (Fig. 4a). This result suggested that reaction system 2 was better for degradation of ZEN. However, with the increase of reaction time, the degradation ratio of ZEN in reaction system 1 reached 41.58% after 10 h of reaction (Fig. 4a). Considering the time cost in actual processing, the reaction system 2 might be the favorable one for degradation of ZEN.

As shown in Fig. 5, the optimal conditions for reaction system 2 were: Tris–HCl buffer (pH 7.5), added with 2.0 mg/mL expressed proteins, reacted at 40 ℃ for 30 min. In compared with the previous studied (Xu 2019), the degradation ratio for ZEN in this work was obviously lower than the results of Xu. The reason for this phenomenon might because of the usage of flexible peptide GGGGS as the linker in the fusion enzyme. As reported previously, usage of the flexible linkers in fusion enzymes might cause dimers of the side-by-side protein domains, and such a conformation obscured the active sites of the fusion enzyme and leading to a decrease in enzyme activity (Chen et al. 2013; Li et al. 2015). In future researches, other types of peptide linkers, such as rigid linkers (EAAAK)_n_, could be tested for elimination of the possible mutual interference of two functional protein domains.

Furthermore, the degradation efficiency of the two mycotoxins in these two systems were still not so good, compared with results of separately studied enzymatic degradation efficiency (Xu 2019; Sun 2020). Reaction systems that are suitable for both enzymes ZHD101.1 and PhcMnp could be further studied in the future.

## Conclusions

Mycotoxins detoxification and degradation are world-wide problems. Although various means had been applied for enhancing degradation efficiency of mycotoxins, there are few reports on fusion enzymes for simultaneous degradation of multiple toxins. For simultaneous degradation of AFB_1_ and ZEN, a fusion enzyme with the functions of zearalenone hydrolase (ZHD101.1) and manganese peroxidase (PhcMnp) was constructed and secretory expressed successfully in this work. The enzymes used for construction of the fusion one were both originated from food safe microorganisms. Meanwhile, the food-grade expression kits, i.e. the host *K. lactis* GG799 and the vector pKLAC1, were used in this work. So the secretory expression of the fusion enzyme ZPF1 was achieved in a food-grade manner. Therefore, this enzyme has the possibility of practical applications in food industry in the future.

### Supplementary Information

**Additional file 1: Table S1.** Primers used in this study for PCR. **Figure S1.** Liquid chromatography of the degradation results of AFB_1_ by the fusion enzyme ZPF1 in reaction system 1. **Figure S2.** Liquid chromatography of the degradation results of ZEN by the fusion enzyme ZPF1 in reaction system 1. **Figure S3.** Liquid chromatography of the degradation results of AFB_1_ by the fusion enzyme ZPF1 in reaction system 2. **Figure S4.** Liquid chromatography of the degradation results of ZEN by the fusion enzyme ZPF1 in reaction system 2.

## Data Availability

All data generated or analyzed during this study are included in this article.

## Abbreviations

AFB_1_: Aflatoxin B_1_
ZEN: Zearalenone
OTA: Ochratoxin A
*K. lactis*: *Kluyveromyces lactis*
*E. coli*: *Escherichia coli*
ZEN-1: Degradation ratio of ZEN by the fusion enzyme ZPF1 in reaction system 1; ZEN-2: degradation ratio of ZEN by the fusion enzyme ZPF1 in reaction system 2
UPLC–MS: Ultra-performance liquid chromatography/tandem mass spectrometry
PCR: Polymerase chain reaction
SDS-PAGE: Sodium dodecyl sulfate polyacrylamide gel electrophoresis
ZPF1: The fusion enzyme constructed by fusing the individual enzymes ZHD101.1 and PhcMnp using the linking peptide GGGGS
