# Supplementary material for: Simultaneous degradation of two mycotoxins enabled by a fusion enzyme in food-grade recombinant Kluyveromyces lactis
Source: Bioresour Bioprocess. 2021 Jul 15;8(1):62. doi: 10.1186/s40643-021-00395-1 (PMC10991947; doi:10.1186/s40643-021-00395-1)
Supplement: Supplementary file 1 — Additional file 1: Table S1. Primers used in this study for PCR. Figure S1. Liquid chromatography of the degradation results of AFB1 by the fusion enzyme ZPF1 in reaction system 1. Figure S2. Liquid chromatography of the degradation results of ZEN by the fusion enzyme ZPF1 in reaction system 1. Figure S3. Liquid chromatography of the degradation results of AFB1 by the fusion enzyme ZPF1 in reaction system 2. Figure S4. Liquid chromatography of the degradation results of ZEN by the fusion enzyme ZPF1 in reaction system 2. [file 40643_2021_395_MOESM1_ESM.docx]

Supplementary Information

**Simultaneous degradation of two mycotoxins enabled by a fusion enzyme in food-grade recombinant *Kluyveromyces lactis***

Yu Xia^1,2,3*^, Zifeng Wu^1,2^, Rui He^1,2^, Yahui Gao^1,2^, Yangyu Qiu^1,2^, Qianqian Cheng^1,2^, Xiaoyuan Ma^1,2,3^, Zhouping Wang^1,2,3^

*Corresponding author: [yuxia@jiangnan.edu.cn](mailto:yuxia@jiangnan.edu.cn)

^1^State Key Laboratory of Food Science and Technology, Jiangnan University, Wuxi 214122, China

^2^School of Food Science and Technology, Jiangnan University, Wuxi 214122, China

^3^Collaborative Innovation Center of Food Safety and Quality Control in Jiangsu Province, Jiangnan University, Wuxi 214122, China

**Table S1** Primers used in this study for PCR.

| Primers | Sequences (5’-3’) |  |
| --- | --- | --- |
| *zhd*101.1-F | CTAGATCTATGAGAACTAGATCAACTATTTC (*Bgl*II) | |
| *zhd*101.1-R | CGTCGACCAAATGTTTTTGAGTAG (*Sal*I) | |
| Phc*mnp*-F | CTAGATCTGGTGGTGGTGGTTCTATGGC (*Bgl*II) | |
| Phc*mnp*-R | CGTCGACTTAAGCTGGACCATC (*Sal*I) | |
| *zhd*101.1-F1-F | CTAGATCTATGAGAACTAGATCAACTATTTC (*Bgl*II) | |
| *zhd*101.1-F1-R | AGCCATAGAACCACCACCACCCAAATG | |
| Phc*mnp*-F1-F | CATTTGGGTGGTGGTGGTTCTATGGCT | |
| Phc*mnp*-F1-R | CGTCGACTTAAGCTGGACCATC (*Sal*I) | |
| *zhd*101.1-F2-F | CTAGATCTATGAGAACTAGATCAACTATTTC (*Bgl*II) | |
| *zhd*101.1-F2-R | AGCCATAGAACCACCACCACCTGAACCACCACCACCCAAATG | |
| Phc*mnp*-F2-F | CATTTGGGTGGTGGTGGTTCAGGTGGTGGTGGTTCTATGGCT | |
| Phc*mnp*-F2-R | CGTCGACTTAAGCTGGACCATC (*Sal*I) | |
| *zhd*101.1-F3-F | CTAGATCTATGAGAACTAGATCAACTATTTC (*Bgl*II) | |
| *zhd*101.1-F3-R | AGCCATAGAACCACCACCACCAGAACCACCACCACCTGAACCACCACCACCCAAATG | |
| Phc*mnp*-F3-F | CATTTGGGTGGTGGTGGTTCAGGTGGTGGTGGTTCAGGTGGTGGTGGTTCTATGGCT | |
| Phc*mnp*-F3-R | CGTCGACTTAAGCTGGACCATC (*Sal*I) | |
| *zhd*101.1-F4-F | CTAGATCTATGAGAACTAGATCAACTATTTC (*Bgl*II) | |
| *zhd*101.1-F4-R | AGCCATAGAACCACCACCACCAGAACCACCACCACCAGAACCACCACCACCTGAACCACCACCACCCAAATG | |
| Phc*mnp*-F4-F | CATTTGGGTGGTGGTGGTTCAGGTGGTGGTGGTTCAGGTGGTGGTGGTTCAGGTGGTGGTGGTTCTATGGCT | |
| Phc*mnp*-F4-R | CGTCGACTTAAGCTGGACCATC (*Sal*I) | |
| pKLAC1-seq-F | ACGGAACCCACACTGGTA | |
| pKLAC1-seq-R | ATCATGCATGTATACATCAG | |

**
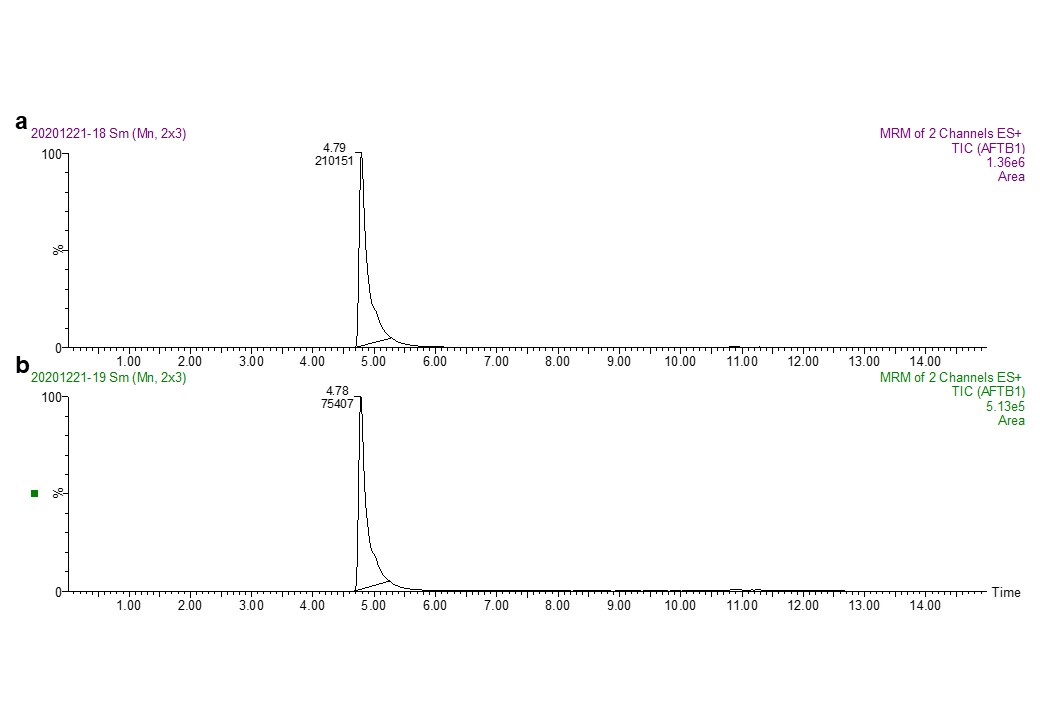
**

**Figure S1** Liquid chromatography of the degradation results of AFB_1_ by the fusion enzyme ZPF1 in reaction system 1. **a** AFB_1_ detected in reaction buffer without addition of enzyme. **b** The residual AFB_1_ detected in degradation results with addition of the fusion enzyme ZPF1.

**
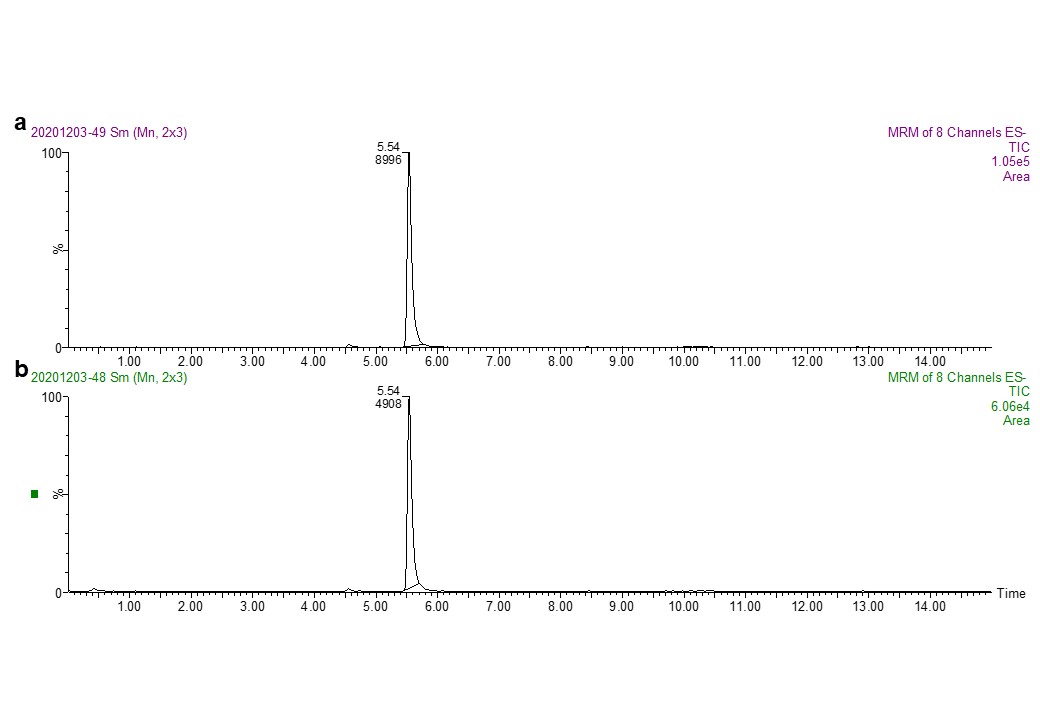
**

**Figure S2** Liquid chromatography of the degradation results of ZEN by the fusion enzyme ZPF1 in reaction system 1. **a** ZEN detected in reaction buffer without addition of enzyme. **b** The residual ZEN detected in degradation results with addition of the fusion enzyme ZPF1.


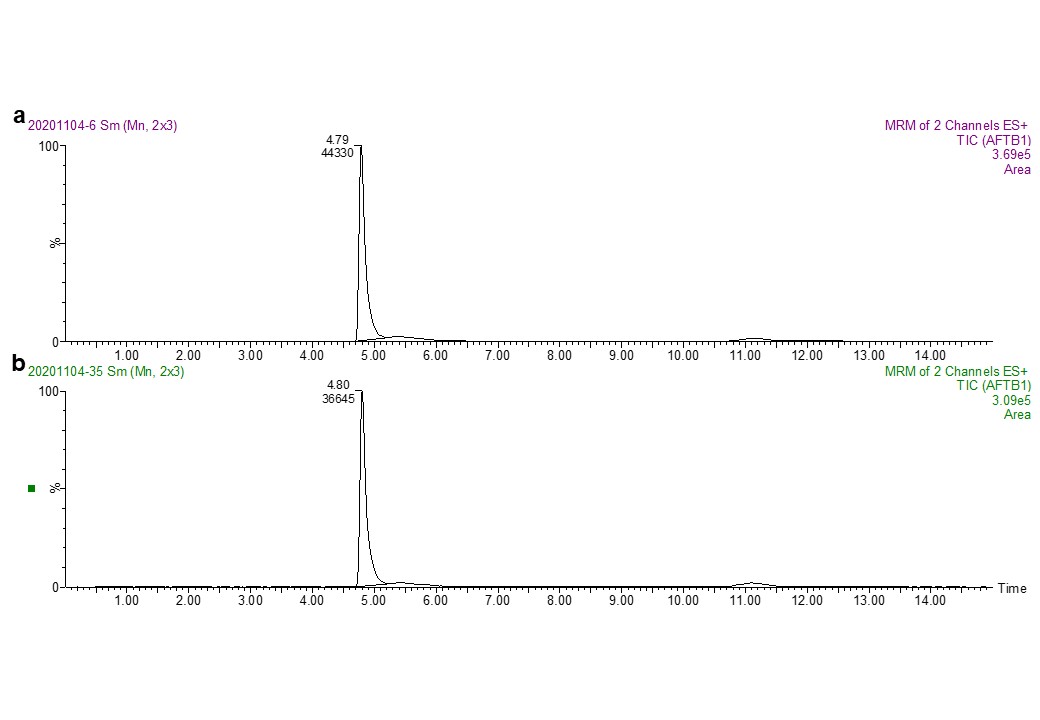


**Figure S3** Liquid chromatography of the degradation results of AFB_1_ by the fusion enzyme ZPF1 in reaction system 2. **a** AFB_1_ detected in reaction buffer without addition of enzyme. **b** The residual AFB_1_ detected in degradation results with addition of the fusion enzyme ZPF1.


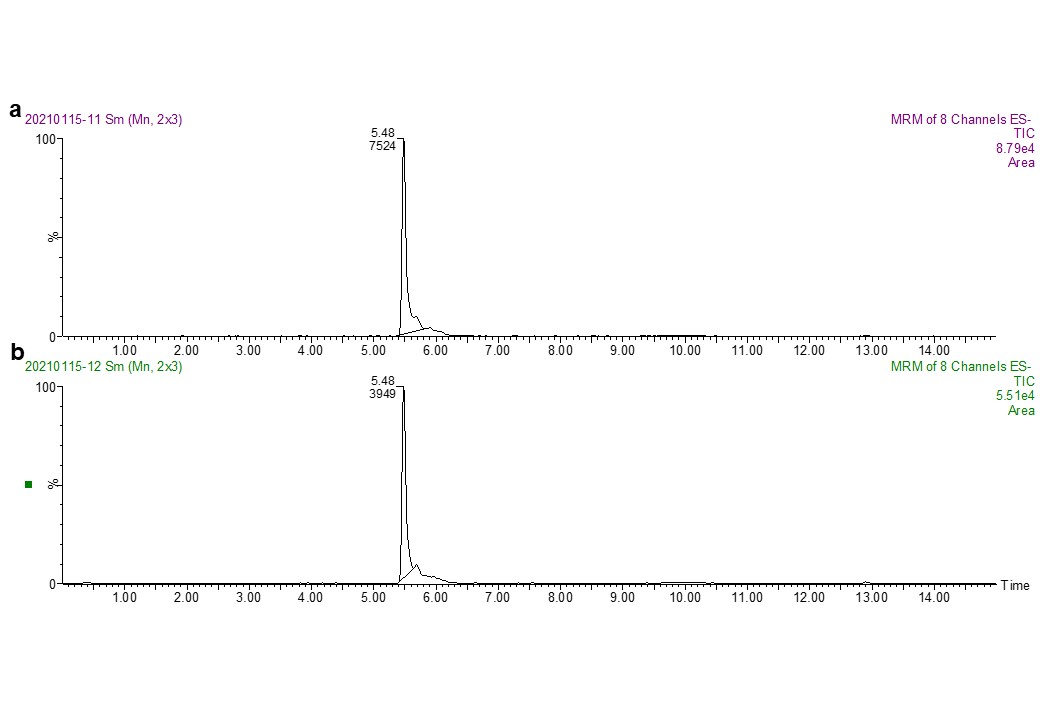


**Figure S4** Liquid chromatography of the degradation results of ZEN by the fusion enzyme ZPF1 in reaction system 2. **a** ZEN detected in reaction buffer without addition of enzyme. **b** The residual ZEN detected in degradation results with addition of the fusion enzyme ZPF1.
